# Hæmaturia Associated with Nephritis

**Published:** 1883-04

**Authors:** Frank V. Walton

**Affiliations:** Lecturer on Anatomy at the Columbia Veterinary College


### IX.—HÆRMATURIA ASSOCIATED WITH NEPHRITIS.

BY FRANK V. WALTON, D.V.S.,

*Lecturer on Anatomy at the Columbia Veterinary College.*

A grey mare was brought to the hospital suffering from discharges of bloody urine.

The previous history of the case was that the animal was kept for driving purposes, and was only used about twice a week, but then was generally driven long distances. Had always been a good feeder, and had not had a sick day for the past five years.

After arriving at the hospital, alternating doses of ergot and ferri perchloridium were administered.

Examination of urine naturally revealed albumen. A microscopic examination was not made, but from the rational symptoms a renal degeneration was suspected.

At the end of five days the hemorrhage stopped, the urine cleared, and the horse was apparently well and sent home.

During the next two weeks she was used for short drives. One day of the third week, while on the road, she was suddenly taken with colicky pains, and was immediately returned to the hospital.

Tympanitis rapidly increased, and the cæcum was punctured to remove the gas, it being the only possible means of relieving the animal and saving life, but this dire resort proved unsuccessful and the mare shortly died.

At the autopsy the principal changes met with were the marked softening and degeneration of the renal tissue The liver also had apparently undergone some granular degeneration. The renal disease was probably the great cause of death.

[We are sorry to have again to call attention to the fact of the incomplete urinary examination, and the entire neglect to examine the kidneys microscopically after removal from the body. Until veterinarians are willing to wake up to more close and accurate work, such as is done in human medicine, they must not expect to advance much from where they now stand, but the very minute they do a mighty change will rapidly follow. By referring to one of the previous cases reported by Dr. Walton you can readily see how positive the diagnosis was before death, and how it was confirmed at the autopsy. By a careful study of these cases we are inclined to the opinion that the line of treatment will finally be such that many cases may be cured which now die at the end of a few days. We know that it takes a little time and trouble to make these accurate diagnosis, but when it will enable the use of the ounce of prevention against a pound of cure it seems quite reasonable to suppose that ultimately it will pay the largest bonus.—ED.]

